# The Construction of an Extreme Radiation-Resistant Perchlorate-Reducing Bacterium Using *Deinococcus deserti* Promoters

**DOI:** 10.3390/ijms252111533

**Published:** 2024-10-27

**Authors:** Shanhou Chen, Zichun Tan, Binqiang Wang, Hong Xu, Ye Zhao, Bing Tian, Yuejin Hua, Liangyan Wang

**Affiliations:** 1 MOE Key Laboratory of Biosystems Homeostasis and Protection, Institute of Biophysics, College of Life Sciences, Zhejiang University, Hangzhou 310058, China; d_wade1982@zju.edu.cn (S.C.); 12407130@zju.edu.cn (Z.T.); wangbinqiang@zju.edu.cn (B.W.); xuhong1685@163.com (H.X.); yezhao@zju.edu.cn (Y.Z.); tianbing@zju.edu.cn (B.T.); 2 Cancer Center, Zhejiang University, Hangzhou 310058, China; 3 Qian Xuesen Collaborative Research Center of Astrochemistry and Space Life Sciences, Ningbo University, Ningbo 315211, China

**Keywords:** dissimilatory perchlorate-reducing bacteria, *Dechloromonas agitata*, *Deinococcus radiodurans*, *Deinococcus deserti*, promoter engineering

## Abstract

Perchlorate is one of the major inorganic pollutants in the natural environment and the living environment, which is toxic to organisms and difficult to degrade due to its special structure. As previously reported, the Phoenix Mars lander detected approximately 0.6% perchlorate in the Martian soil, indicating challenges for Earth-based life to survive there. Currently, biological approaches using dissimilatory perchlorate-reducing bacteria (DPRB) are the most promising methods for perchlorate degradation. However, the majority of DPRB exhibit limited radiation resistance, rendering them unsuitable for survival on Mars. In this study, we obtained the transcriptome data of *Deinococcus deserti*, and predicted and identified multiple constitutive expression promoters of *D*. *deserti* with varying activities. The top-five most active promoters were separately fused to specific genes involved in the degradation of perchlorate from DPRB *Dechloromonas agitata* CKB, and transformed into *Deinococcus radiodurans* R1, forming a novel dissimilatory perchlorate-reducing bacterium, R1−CKB. It exhibited both efficient perchlorate degradation capability and strong radiation resistance, potentially offering a valuable tool for the further enhancement of the Martian atmosphere in the future.

## 1. Introduction

Perchlorate can be used as a rocket fuel [1], an oxidizer in fireworks [2], and an explosive in airbag systems [3]. It is one of the major inorganic pollutants in the natural environment and is abundant in surface water, groundwater, soil, many food and drinking water sources [4,5,6]. As an endocrine disruptor, perchlorate ion has a similar radius and charge as iodide ion, which competes with iodide to enter the thyroid gland when it enters the human body, interfering with the absorption of iodide [7,8]. Due to its tetrahedron structure, perchlorate exhibits high chemical stability and excellent migration capabilities [9], rendering its removal challenging.

Currently, technologies for degrading perchlorate include physical methods, chemical methods, and biological methods. [10]. Among them, biological methods are the most promising method for perchlorate degradation. Dissimilatory perchlorate-reducing bacteria (DPRB) are widely present in the natural environment, with isolated strains mainly belonging to *Proteobacteria*, including α-*Proteobacteria*, β-*Proteobacteria*, γ-*Proteobacteria* and ε-*Proteobacteria* [11]. The pathway of perchlorate degradation by DPRB is as follows: initially, perchlorate is degraded to chlorate by perchlorate reductase, then further degraded to chlorite, and ultimately reduced to chloride ion and oxygen by chlorite dismutase. Perchlorate reductase is encoded by the operon *pcrABCD*, with PcrAB being the effective catalytic part of perchlorate reductase encoded by *pcrA* and *pcrB*, PcrC being responsible for electron transfer encoded by *pcrC*, and PcrD being responsible for the assembly and modification of the PcrAB protein complex encoded by *pcrD*. Chlorite dismutase is encoded by the gene *cld* [12,13,14,15].

Not only does perchlorate exist on Earth, but traces of it have also been found on Mars [16,17,18]. As previously reported, the Phoenix Mars lander detected approximately 0.6% perchlorate in the Martian soil [19]. High concentrations of perchlorate could have a significant toxic effect on living organisms, posing a major challenge for life on Mars. Due to the extreme Martian environment with high levels of radiation, most DPRB have poor radiation resistance and are unable to survive on Mars. Therefore, they cannot provide direct and effective assistance for improving the future Martian atmosphere.

As is well known, *Deinococcus radiodurans* is one of the most radiation-resistant organisms on Earth, boasting a unique DNA damage repair system [20,21,22]. Integrating certain genes involved in the degradation of perchlorate into *D. radiodurans* could potentially create a novel dissimilatory perchlorate-reducing bacterium that possesses both efficient perchlorate degradation ability and strong radiation resistance. To ensure the successful expression of the cloned genes within *D. radiodurans*, it is necessary to insert universal promoters for *D. radiodurans* upstream of each gene. The strength of a promoter is determined by the specificity of RNA polymerase σ factor for different promoter sequences, and therefore most promoters cannot be universally used [23]. To optimize the expression of target genes, the genetic modification of promoters is often required to achieve controllable regulation at the gene level. This can be achieved through the following two main strategies: the mutation of endogenous promoters, and the replacement of endogenous or exogenous promoters [24,25,26].

*Deinococcus deserti* also belongs to the *Deinococcus* genus and has a certain genetic distance from *D. radiodurans* [27,28,29], whose heterologous promoter expression is less affected by endogenous repressors in *D. radiodurans*, making its transcriptome an excellent resource for screening constitutive promoters of *D. radiodurans*. In this study, we predicted the promoter sequences of *D. deserti* using bioinformatics analysis based on the transcriptome data of *D. deserti* [30], and a series of test vectors for constitutive promoter elements were constructed. With *D. radiodurans*’ classic strong promoter, the *groES* promoter [31,32], serving as the reference benchmark, we identified multiple constitutive promoters of *D. deserti* with varying activities. The top-five most active promoters were separately inserted into certain genes involved in the degradation of perchlorate (*cld*, *pcrA*, *pcrB*, *pcrC*, *pcrD*) from DPRB *Dechloromonas agitata* CKB, and transformed into *D. radiodurans* R1 to obtain a novel dissimilatory perchlorate-reducing bacterium R1−CKB with proficient perchlorate degradation and robust radiation resistance.

## 2. Results

### 2.1. Prediction of Strong Promoters from D. deserti

**Figure 1:**
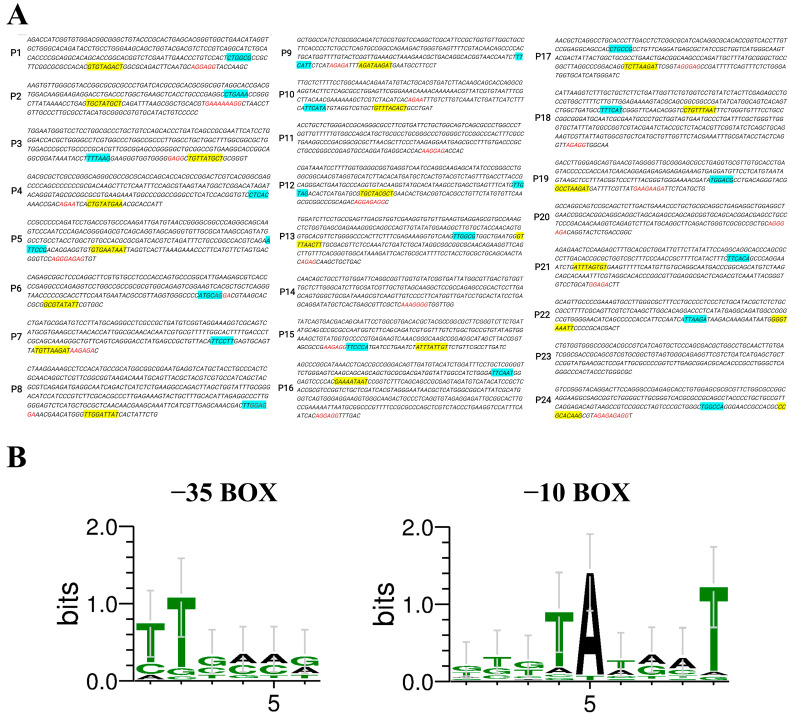
(**A**) Predicted promoters and their sequence compositions. The −35 box is labeled in blue, the −10 box is labeled in yellow, and the RBS is labeled in red. (**B**) Conserved base analysis of the predicted promoter’s −35 box and −10 box.

The top 24 genes (temporarily named gene_1 to gene_24), as identified from the transcriptome data of *D. deserti* [30] and ranked by their FPKM values, are listed in Table 1. The predicted promoter regions were designated as P1–P24, corresponding to their respective genes. The −35 box, −10 box regions, and putative ribosome binding sites (RBSs) of the promoters were identified and labeled using different colors, as shown in Figure 1A. The specific promoter sequences of P1–P24 were listed in Table S1. Among these promoters, the −35 box of P11, P14, P20, and P23 could not be predicted, suggesting that their promoter activities might be weak.

Multiple sequence alignment was performed on the −35 box and −10 box of the predicted promoters, and the patterns were summarized, as depicted in Figure 1B. The −35 box center is typically located around −35 bp upstream of the start codon, containing signals recognized by RNA polymerase, and the statistical frequency of each base is T_82_T_84_G_78_A_65_C_54_A_45_ [33]. The −10 box is a conserved sequence located around −10 bp upstream of the start codon, containing many A-T base pairs that facilitate the local separation of duplex DNA strands, and the statistical frequency of each base is T_80_A_95_T_45_A_60_A_50_T_96_ [33]. The alignment results obtained from our analyses were consistent with the statistical frequencies of prokaryotic promoter sequences.

### 2.2. Assessment of D. deserti Promoter Activities in D. radiodurans

The activities of each promoter element were assessed by measuring the transcriptional and translational efficiency with the reporter gene *mCherry* in *D. radiodurans*. Among them, the promoter elements carrying P4, P12, and P24 failed to be transformed into *D. radiodurans*, and the growth curves of the remaining promoter elements were tested, as shown in Figure S1.

**Figure 2:**
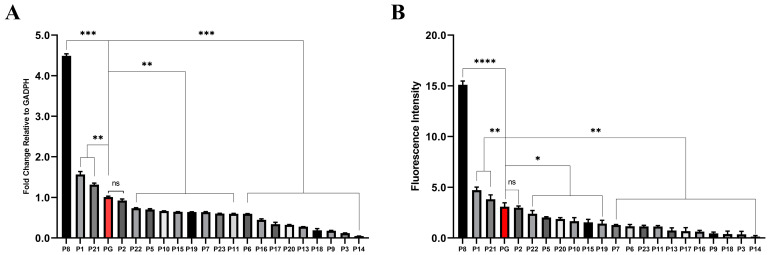
Comparative analysis of the transcriptional (**A**) and translational (**B**) levels of the reporter gene *mCherry* in each transformed strain. PG (marked in red) is the control promoter (* *p* < 0.05, ** *p* < 0.01, *** *p* < 0.001, **** *p* < 0.0001, ns: not significant).

The transcriptional levels were analyzed using RT-qPCR and calculated by the 2^−ΔΔct^ method, with the *D. radiodurans*’ classic strong promoter, *groES* promoter [31,32], serving as the control reference (pRAD-PG). As shown in Figure 2A, the transcriptional levels of the reporter genes in pRAD-P8, pRAD-P1, and pRAD-P21 possessed relatively strong activities, approximately 4.5, 1.5, and 1.3 times that of pRAD-PG, respectively. The transcription level of pRAD-P2 was equivalent to that of pRAD-PG, while the transcription levels of the other pRAD-promoters were slightly or much lower than that of pRAD-PG. 

The translational levels of the reporter gene *mCherry* were further investigated using a fluorescence intensity ratio method. The non-transformed *D. radiodurans*’ deletion strain Δ*dr0862* was used as a blank control, which was to exclude the interference of pigments in *D. radiodurans* on the detection of the expression component strength. As shown in Figure 2B, the fluorescence intensity value of pRAD-PG was 3.1, while the fluorescence intensities of pRAD-P8, pRAD-P1, and pRAD-P21 were approximately 5.0, 1.6, and 1.3 times that of pRAD-PG, respectively. The fluorescence intensity of pRAD-P2 was comparable to that of pRAD-PG, whereas the levels of the other pRAD promoters were marginally lower than that of pRAD-PG, which align with their transcriptional levels. 

Based on the results above, the promoter activities were classified, and the results were shown in Table S2. The top-five most active promoters (P8, P1, P21, P2, P22) were selected for subsequent experiments.

### 2.3. Expression of the Target Genes in D. radiodurans 

We cloned the perchlorate-reducing related genes (*cld*, *pcrA*, *pcrB*, *pcrC*, *pcrD*) from DPRB *D. agitata* CKB and separately fused to the selected promoters, as indicated in Figure S2. The engineered recombinant vector was transformed into *D. radiodurans* R1, resulting a novel bacterium designated as R1−CKB.

**Figure 3:**
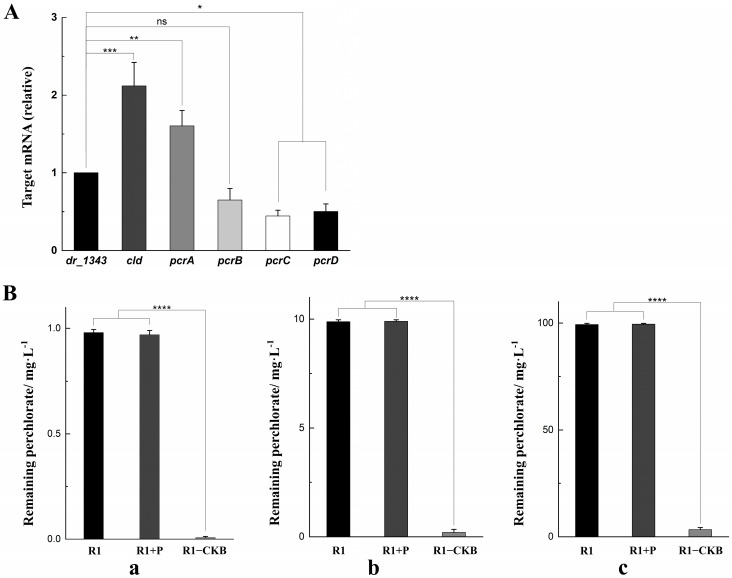
(**A**) The transcription levels of the target genes compared to *dr_1343*. (**B**) The residual perchlorate in each degradation funnel. (**a**) 1 mg/L sodium perchlorate, 1 mg/L sodium acetate, 50 μg/mL hemin. (**b**) 10 mg/L sodium perchlorate, 10 mg/L sodium acetate, 50 μg/mL hemin. (**c**) 100 mg/L sodium perchlorate, 100 mg/L sodium acetate, 50 μg/mL hemin (* *p* < 0.05, ** *p* < 0.01, *** *p* < 0.001, **** *p* < 0.0001, ns: not significant).

The relative transcription levels of the target genes in R1−CKB were shown in Figure 3A. The housekeeping gene *dr_1343*, which encodes glyceraldehyde-3-phosphate dehydrogenase, is commonly used for the normalization of mRNA expression levels [34,35,36]. Given that *dr_1343* typically exhibits stable high expression in *D. radiodurans*, it is evident that all the transcription levels of the target genes were relatively high.

In addition, the R1−CKB experimental group, the R1 control group, and the R1 + P group (which was a control group including a control strain containing the promoters but without the target genes) were each placed in the funnels for the perchlorate degradation assay (as described in the Materials and Methods Section 4.6), with the perchlorate concentrations being quantified by the solvent extraction method. The perchlorate content in each funnel was subsequently measured using spectrophotometric determination [37], and the concentrations of the residual perchlorate in each funnel were depicted in Figure 3B. In cultures of strain R1, the remaining perchlorate concentrations were 0.98 mg/L, 9.88 mg/L, 99.31 mg/L, respectively. Similarly, in group R1 + P, the remaining perchlorate concentrations were 0.99 mg/L, 9.92 mg/L, 99.47 mg/L, respectively. In contrast, strain R1−CKB displayed significantly lower residual perchlorate concentrations, with the values of only 0.008 mg/L, 0.21 mg/L, 3.32 mg/L, respectively. It was evident that strain R1−CKB exhibited a strong ability of degrading perchlorate.

### 2.4. Stress-Resistant Phenotypes of R1−CKB

**Figure 4:**
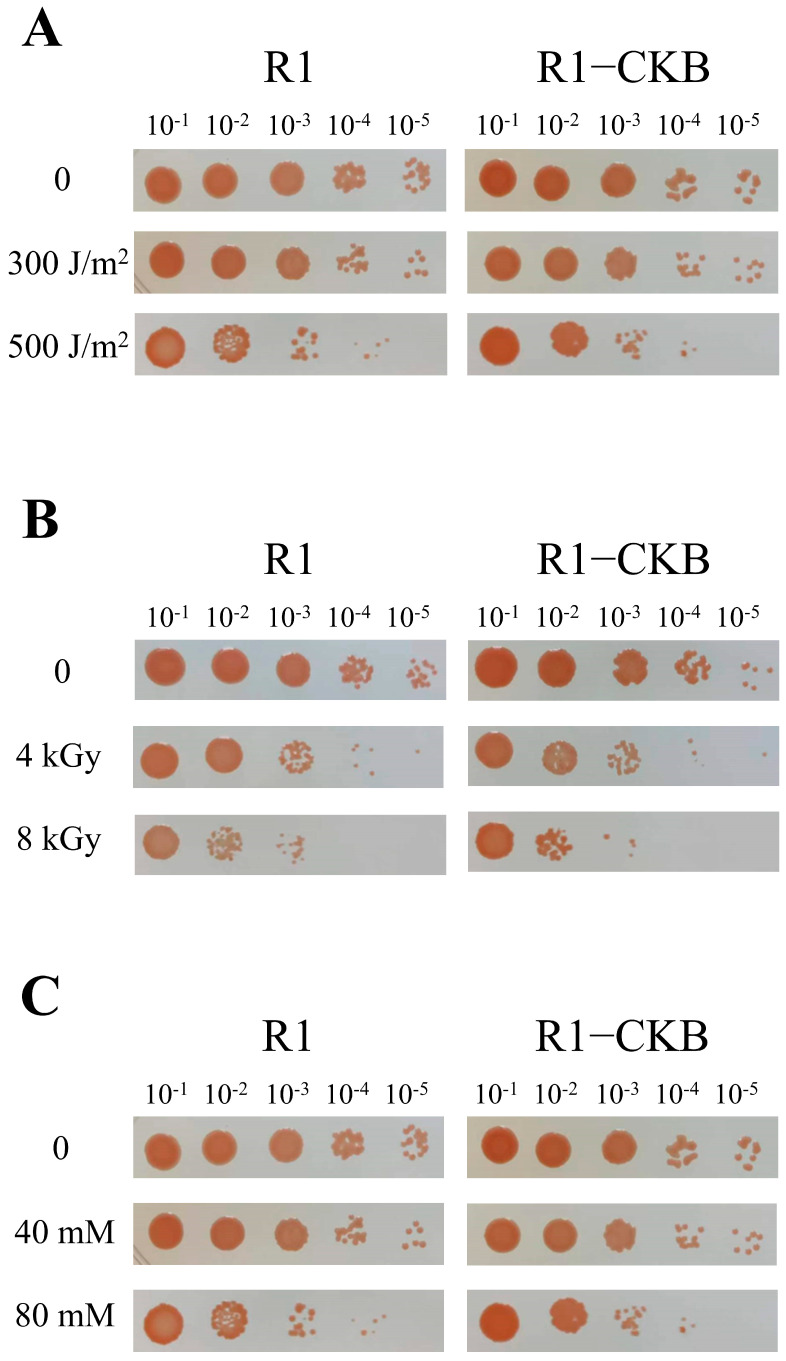
Phenotypes of R1 and R1−CKB following stress treatments: (**A**) UV irradiation (0, 300, 500 J/m^2^). (**B**) Gamma radiation (0, 4, 8 kGy). (**C**) H_2_O_2_ oxidative stress (0, 40, 80 mM) for 30 min.

The recombinant strain R1−CKB and the wild strain R1 were each exposed to various stress treatments, including UV irradiation, gamma radiation and oxidative stress induced by H_2_O_2_. The post-treatment survival rates were depicted in Figure 4. Similar to strain R1, strain R1−CKB also exhibited robust resistance to various agents, enduring high doses of UV irradiation, gamma radiation, as well as high concentrations of H_2_O_2_, demonstrating that the perchlorate-reducing bacterium R1−CKB possessed exceptional radiation resistance.

## 3. Discussion

It is well known that the extreme environmental tolerance and unique genetic resources of *D. radiodurans* provide important foundations for synthetic biology studies, offering crucial insights into applications such as enhancing organism resilience and productivity traits [38]. Unfortunately, investigations into biological components of *D. radiodurans* are scarce compared to those on model organisms such as *E. coli* and *Lactobacillus* [39,40,41], particularly regarding the development of exogenous promoter libraries for *D. radiodurans*.

FPKM values were commonly used for characterizing promoters [42,43], which were also utilized in this study to screen out 24 potential highly transcribed genes from the transcriptome of *D. deserti*. The promoter sequences of these genes were effectively cloned in vitro and integrated into the transformation vector pRAD plasmid, facilitating the development of a testing vector for constitutive gene-expression promoter elements. By introducing the testing vector into *D. radiodurans*, the characterization and identification of multiple promoters with varying strengths were achieved. The results indicated that P8, P1, and P21 are strong promoters; P2 has activity like PG; P5–P7, P10, P11, P15, P19, P22, and P23 are relatively weak promoters; P13, P16, and P17 are weak promoters; and P3, P9, P14, and P18 are very weak promoters.

Native *D. radiodurans* promoters were identified and utilized for tunable gene expression in *D. radiodurans* by Chen et al. [44], in which the *groES* promoter was also used as a reference benchmark. Compared to the *groES* promoter, the relative fluorescence intensities of the promoter elements in this study were, on average, stronger than those reported in their work, as indicated in Figure S3. Notably, the most active promoter identified in this study, P8, exhibited significantly greater strength compared to the strongest promoters, *PDR_1261* and *PrpmB*, as concluded by Chen et al. Furthermore, the concurrent introduction of additional promoters from *D. radiodurans* may result in excessive repetitive sequences within its genome, potentially leading to genomic instability over time [45], not to mention its inherent robust recombination capability [46]. Therefore, it might be a more stable way to select promoters from *D. deserti*, which has a certain genetic distance from *D. radiodurans*, resulting in less interference by endogenous repressors on heterologous promoter expression in *D. radiodurans*. Thus, this study broadened the range of promoters accessible for *D. radiodurans*, culminating in a selection of resilient promoters with diverse strengths and characteristics that hold potential for a wide array of applications.

In addition, we selected the top-five most robust promoters from the aforementioned set, which were fused with certain genes involved in the degradation of perchlorate (*cld*, *pcrA*, *pcrB*, *pcrC*, *pcrD*) from DPRB *D. agitata*, and transformed into *D. radiodurans* to create a novel dissimilatory perchlorate-reducing bacterium R1−CKB. This strain not only exhibited a strong ability of degrading perchlorate, but also demonstrated a strong resistance to extreme conditions, such as high doses of UV irradiation, high doses of gamma radiation, and high concentrations of H_2_O_2_ oxidative stress. While most radiation-resistant bacteria cannot reduce perchlorate and most DPRB would not survive radiation stress, the strain R1−CKB might become an ideal microorganism for exploring Mars in the future. BLAST was employed to identify the homologous genes in *D. radiodurans* corresponding to the top-five promoters, with gene_1 corresponding to *dr_1942*, gene_2 to *dr_2330*, and gene_8 to *dr_2389*; however, homologous genes for gene_21 and gene_22 were not identified. Subsequently, we compared these homologs with the transcriptome data from *D. radiodurans* under radiation stress [47] and observed the significant activation of all three homologous genes following irradiation. Therefore, we speculate that radiation stress may activate these promoters, or at the very least, may not inhibit their activities. If confirmed, this would serve as a positive indicator for R1−CKB, which may need to function in a radiation environment for an extended period. Additionally, it is worth mentioning that if R1−CKB is indeed widely used on Martian soil in the future, the ethical considerations and practical challenges associated with deploying genetically modified organisms in extraterrestrial environments should be taken into account. Measures in compliance with the Outer Space Treaty need to be implemented, such as isolating R1−CKB and culturing it within a controlled environment.

## 4. Materials and Methods

### 4.1. Bioinformatic Analysis of the D. deserti Transcriptome

We obtained transcriptome data of *D. deserti* under normal growth conditions with the NCBI SRA accession number SRX2611096 [48], calculated the Fragments Per Kilobase Million (FPKM) values representing gene expression intensity, and sorted them. BLAST was used to determine the gene accession numbers and functional annotations corresponding to the transcriptome accession number. Based on the FPKM values, we temporarily named the top 24 genes from high to low gene 1–gene 24. Then, 500 bp upstream regions of the 24 predicted highly expressed genes from Table 1 were selected, and the online promoter prediction tool BPROM (SoftBerry, http://www.softberry.com/berry.phtml?topic=bprom&group=programs&subgroup=gfindb, accessed on 23 October 2024) was used to analyze the sequences and structures of their promoters. The predicted promoter regions were designated as P1–P24, and primers used to amplify P1–P24 were listed in Table S3.

### 4.2. Strains, Plasmids, and Culture Conditions

*D. radiodurans* and its derivatives were cultivated in TGY medium (0.5% tryptone, 0.1% glucose, and 0.3% yeast extract) at 30 °C, shaking at 200 rpm. *D. agitata* was cultivated in R2A medium (0.05% proteose peptone, 0.05% casamino acids, 0.05% yeast extract, 0.05% dextrose, 0.05% soluble starch, 0.03% dipotassium phosphate, 0.005% magnesium sulfate, and 0.03% sodium pyruvate) at 30 °C, shaking at 200 rpm. *E. coli* DH5α was used as the host strain for the construction of recombinant plasmids and cultured at 37 °C in LB medium (1% tryptone, 0.5% yeast extract, and 1% NaCl). When necessary, antibiotics were added at a final concentration of 100 μg/mL ampicillin for *E. coli*, and 4 μg/mL chloramphenicol for *D. radiodurans*.

### 4.3. DNA Manipulation and Plasmid Construction

The promoter-activity testing vector was constructed by enzymatic ligation, as shown in Figure S4. In the PCR amplification reaction, restriction endonucleases *Sac*I and *Spe*I were used for P4 and P22, while *Hind*III and *Spe*I were used for the others. The double digestion was performed overnight at 37 °C. The resulting promoter fragments with sticky ends were ligated with the linear plasmid vector pRAD-*mCherry* (lab stock) using T4 DNA ligase at 16 °C overnight. The obtained plasmid was transformed into 500 μL competent cells of *D. radiodurans* deletion strain Δ*dr0862* (lab stock), which was to exclude the interference of pigments in *D. radiodurans* on the detection of the expression component strength, and incubated on ice for 30 min. The mixture was then transformed into 5 mL of TGY medium and cultivated at 30 °C, shaking at 200 rpm overnight. After that, 200 μL of the mixture was spread onto a TC_4_ agar plate (TGY agar containing 4 μg/mL chloramphenicol), and then incubated at 30 °C for about 5 days until the colonies were visible.

The target genes (*cld, pcrA, pcrB, pcrC, pcrD*) were amplified with homologous arms, and inserted into the linear plasmid vector pRAD-*mCherry* through homologous recombination. After that, the top-five most active promoters (P8, P1, P22, P2, P21) were also amplified with homologous arms, and inserted into the linear plasmid vector through homologous recombination one by one. The primers used for homologous recombination were listed in Table S4, and the constructed target fragment was shown in Figure S2. The plasmid transformation method mirrored the one described above, and the obtained colonies were designated as R1−CKB.

### 4.4. Real-Time Quantitative PCR (RT-qPCR)

Strains were cultivated until OD_600_ = 1.0, and then 1 mL of the culture was centrifuged at 3000× *g* for 3 min. The supernatant was discarded and 200 μL of lysozyme solution (dissolved in DEPC-treated water) was added to the pellet. It was incubated at 37 °C for 30 min to lyse the cells. Total RNA was extracted from the suspension using a TransZol Up Plus RNA Kit (TransGen, Beijing, China) according to the manufacturer’s instructions. For real-time quantitative PCR analysis (RT-qPCR), cDNA was synthesized from 1 μg of total RNA using a HiScript III 1st Strand cDNA Synthesis Kit (+gDNA wiper) (Vazyme, Nanjing, China) following the manufacturer’s instructions. RT-qPCR amplification was conducted using the TB Green^®^ Fast qPCR Mix (Takara, Tokyo, Japan) on an Mx3005p (Stratagene, San Diego, CA, USA).

### 4.5. The Detection of Protein Fluorescence Intensity

Single colonies were picked from the transformation strain plate and cultured to OD_600_ = 1.0, and then 300 μL of each was taken and transferred to a 96-well black plate. The non-transformed *D. radiodurans* deletion strain Δ*dr0862* was used as a blank control. The mCherry fluorescence intensity of each transformed strain was captured by exciting it at a wavelength of 488 nm and absorbing it at 588 nm using a SpectraMax M5 Multi-Mode Microplate Reader (Molecular Devices, San Jose, CA, USA), aiming to ascertain the expression level of the reporter protein in each transformation.

### 4.6. The Determination and Degradation of Perchlorate

The perchlorate was determined using the solvent extraction method [37]. After the 4.0 × 10^−4^ M methylene blue solution and the standard perchlorate solution were prepared, 10.0 mL of the standard perchlorate solution (0.25 mg/L, 0.5 mg/L, 0.75 mg/L, and 1.0 mg/L, respectively) was placed in a separatory funnel. Then, 0.5 mL of 0.05 M sulfuric solution and 10.0 mL of dichloroethane were added. The separatory funnel was shaken for about 30 s, whereby the complex formed between methylene blue and perchlorate was extracted into the organic layer. When the two layers had clearly separated, the organic layer was transferred to a glass tube with a glass stopper, about 0.5 g of the anhydrous sodium sulfate was added, and the mixture was shaken vigorously to make it transparent. With distilled water serving as the reference, the absorbance of the clear solution was measured at 655 nm, using a BioSpectrometer (Eppendorf, Hamburg, Germany).

*D. radiodurans* R1, R1 + P, and R1−CKB were cultivated in 100 mL TGY culture media at 30 °C, shaking at 200 rpm until the OD_600_ = 1.0. Then, the following three treatments were performed: (A) 1 mg/L sodium perchlorate, 1 mg/L sodium acetate, and 50 μg/mL hemin. (B) 10 mg/L sodium perchlorate, 10 mg/L sodium acetate, and 50 μg/mL hemin. (C) 100 mg/L sodium perchlorate, 100 mg/L sodium acetate, and 50 μg/mL hemin. After that, all the treatments were separately put into Oxoid AnaeroJar 2.5 L anaerobic jars (Thermo Fisher, Waltham, MA, USA), respectively, and anaerobic gas packs AnaeroPack^TM^-Anaero (Mitsubishi Gas Chemical, Tokyo, Japan) were added. The samples were incubated at 30 °C for 30 days after sealing the jars.

### 4.7. The Phenotypes of R1 and R1−CKB

UV irradiation treatment was carried out as follows: *D. radiodurans* and R1−CKB were cultivated in 5 mL TGY culture media at 30 °C, shaking at 200 rpm until the OD_600_ = 1.0. Then, 1 mL of bacterial solution was collected and centrifuged at 3000× *g* for 3 min, the supernatant was discarded, and the bacterial cells were washed with 1×PBS solution once. Then, 1 mL 1×PBS solution was used for resuspending the bacterial cells, and the bacterial solutions were serially diluted 10-fold with 1×PBS solution to obtain dilutions of 10^1^, 10^2^, 10^3^, 10^4^, and 10^5^. Then, 5 μL of each dilution was spotted onto TGY agar media and irradiated with UV radiation at doses of 0, 100, 200, 300, 400, and 500 J/m^2^. The plates were incubated upside down at 30 °C for approximately 2 days.

γ radiation treatment was carried out as follows: The pretreatment for γ radiation was similar to that for UV irradiation, except that after washing the bacterial cells once, they were not resuspended in solution but directly irradiated by ^60^Co with doses of 0, 2, 4, 6, 8, and 10 kGy. After the radiation, 1 mL 1×PBS solution was used for resuspending the bacterial cells, and the bacterial solutions were serially diluted 10-fold with 1×PBS solution to obtain dilutions of 10^1^, 10^2^, 10^3^, 10^4^, and 10^5^. Then, 5 μL of each dilution was spotted onto TGY agar. The plates were incubated upside down at 30 °C for approximately 2 days.

H_2_O_2_ oxidative stress was carried out as follows: The pretreatment for H_2_O_2_ oxidative stress was like that for UV irradiation. After resuspending the bacterial cells, H_2_O_2_ was added to the solution at final concentrations of 0, 20, 40, 60, 80, and 100 mM, and then the mixture was reacted at room temperature for 30 min. The reaction was terminated by adding catalase at a final concentration of 20 ng/μL and incubating for 10 min. Then, the bacterial solutions were serially diluted 10-fold with 1×PBS solution to obtain dilutions of 10^1^, 10^2^, 10^3^, 10^4^, and 10^5^, and 5 μL of each dilution, which were spotted onto TGY agar media. The plates were incubated upside down at 30 °C for approximately 2 days.

## Figures and Tables

**Table 1:** The top 24 highly abundant genes and their FPKM values.

Name	Transcript ID	Gene ID	Protein Characteristics	FPKM
gene_1	RS03290	Deide_05740	acyl carrier protein	27,414.9
gene_2	RS12670	Deide_22131	putative ferredoxin	20,881.3
gene_3	RS19070	Deide_17971	hypothetical protein	19,113.8
gene_4	RS19355	Deide_04426	conserved hypothetical protein, precursor	13,246.9
gene_5	RS02555	Deide_04493	hypothetical protein	13,108.6
gene_6	RS10680	Deide_18600	putative Thioredoxin	11,626.1
gene_7	RS11895	Deide_20641	hypothetical protein	8012.08
gene_8	RS00720	Deide_01280	putative transglycosylase associated protein	6118.36
gene_9	RS00860	Deide_01461	putative adenylate/guanylate cyclase	5842.14
gene_10	RS13035	Deide_22810	hypothetical protein	5471.95
gene_11	RS10440	Deide_18130	hypothetical protein	5034.39
gene_12	RS10375	Deide_18000	hypothetical protein	4916.47
gene_13	RS19380	Deide_15270	hypothetical protein	4738.24
gene_14	RS01615	Deide_02853	hypothetical protein	4392.33
gene_15	RS06335	Deide_11052	preprotein translocase SecG subunit	3790.97
gene_16	RS11225	Deide_19564	hypothetical protein	3758.24
gene_17	RS04270	Deide_07352	hypothetical protein	3715.04
gene_18	RS18505	Deide_20313	hypothetical protein	3694.73
gene_19	RS03580	Deide_06180	conserved hypothetical protein, precursor	3349.25
gene_20	RS08665	Deide_15010	putative Peptidylprolyl isomerase	3184.30
gene_21	RS01005	Deide_01740	putative peptidase S8 and S53	3099.67
gene_22	RS03375	Deide_05864	hypothetical protein	3080.87
gene_23	RS19395	Deide_19231	hypothetical protein	2953.04
gene_24	RS01030	Deide_01780	conserved hypothetical protein	2907.72

## Data Availability

All data that support the findings of this study are included within the manuscript (and any Supplementary Information Files).

## Supplementary Materials

The following supporting information can be downloaded at: https://www.mdpi.com/article/10.3390/ijms252111533/s1.
