# Supplementary material for: The Construction of an Extreme Radiation-Resistant Perchlorate-Reducing Bacterium Using Deinococcus deserti Promoters
Source: Int J Mol Sci. 2024 Oct 27;25(21):11533. doi: 10.3390/ijms252111533 (PMC11546323; doi:10.3390/ijms252111533)
Supplement: Supplementary file 1 [file ijms-25-11533-s001.zip › ijms-3259897-supplementary.pdf]

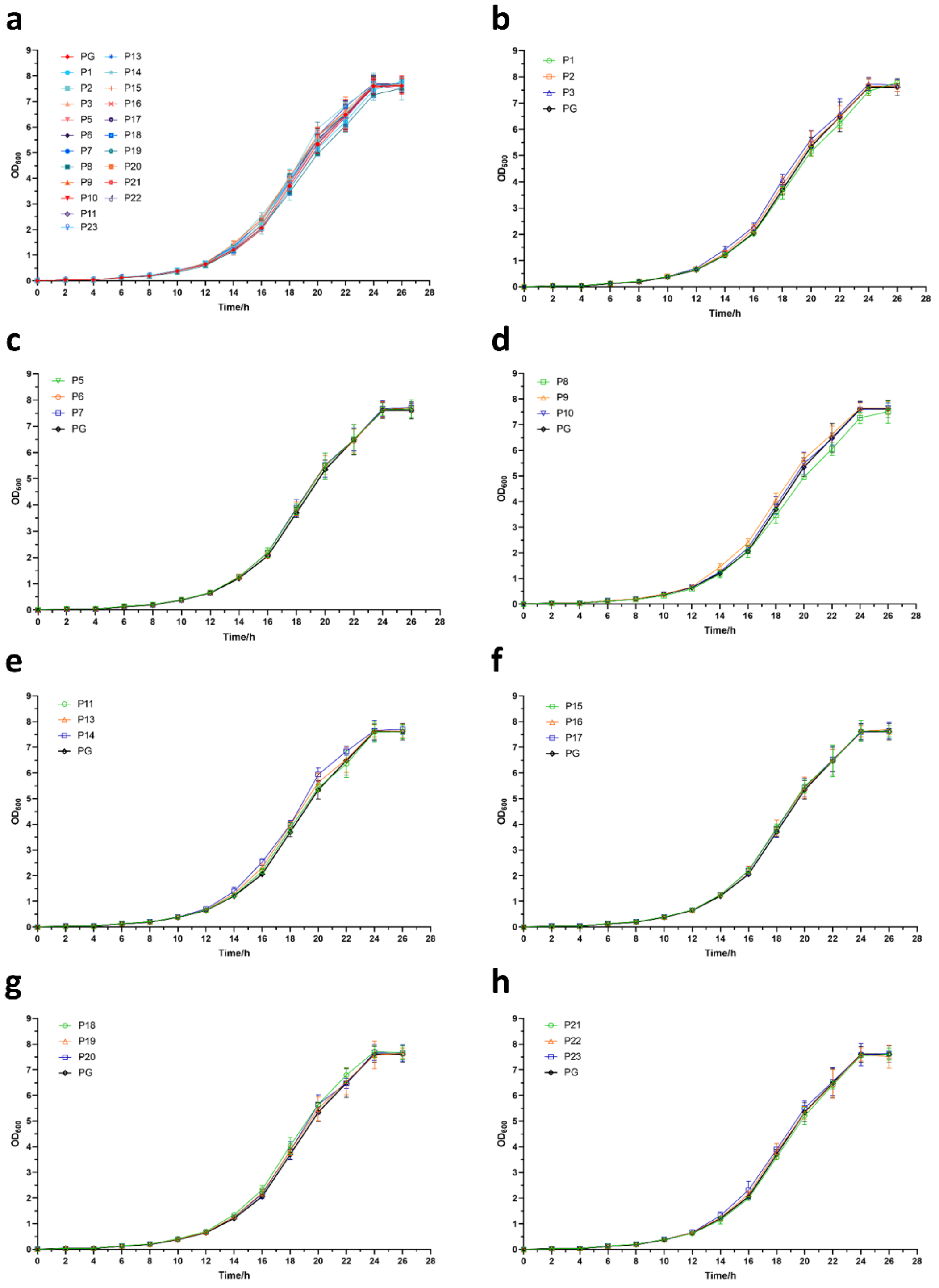

Figure S1. The growth curves of each promoter elements. **(a)** PG, P1−P3, P5−P11, P13−P23. **(b)** PG, P1, P2, P3. **(c)** PG, P5, P6, P7. **(d)** PG, P8, P9, P10. **(e)** PG, P11, P13, P14. **(f)** PG, P15, P16, P17. **(g)** PG, P18, P19, P20. **(h)** PG, P21, P22, P23.

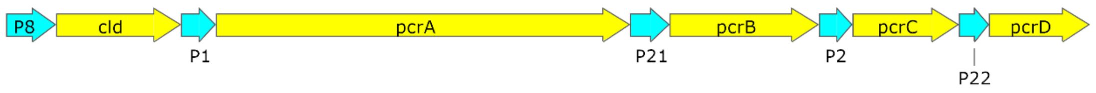

Figure S2. The constructed target fragment containing the target genes and five promoters.

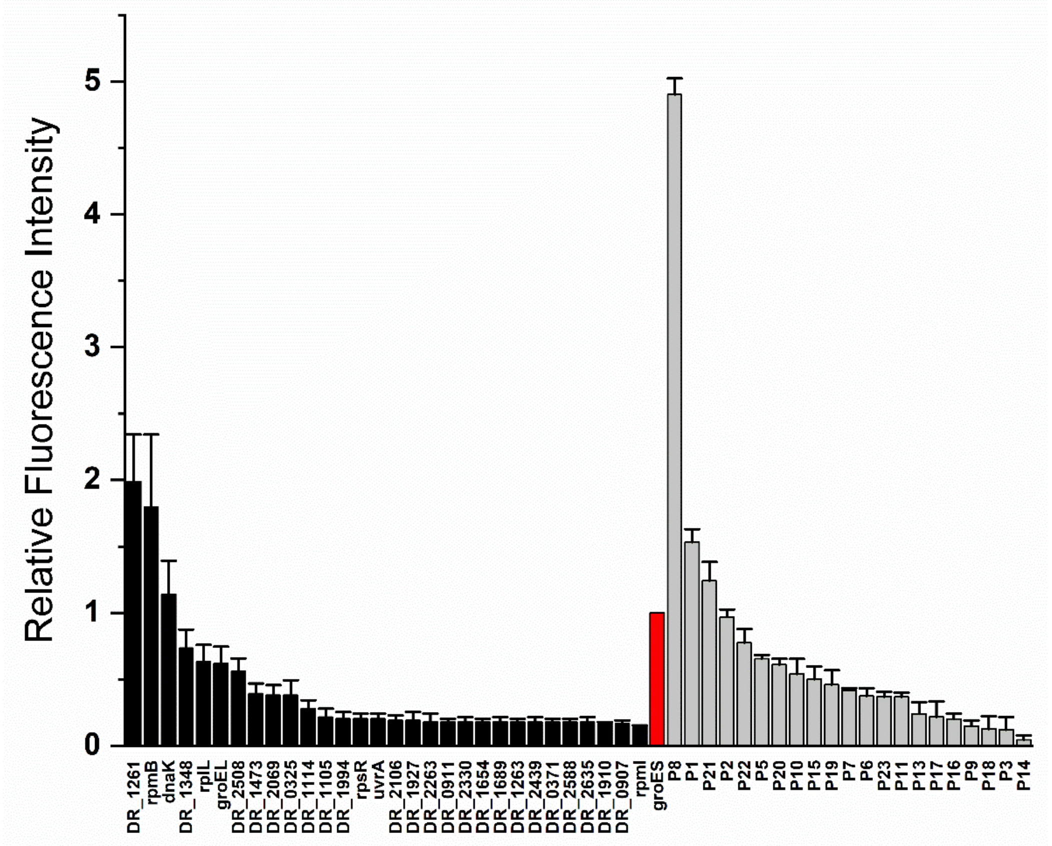

Figure S3. The relative fluorescence intensities of the promoter elements compared to the *groES* promoter element. The promoter elements identified by Chen et al. were marked in black, whereas those in this study were highlighted in gray. Additionally, the reference benchmark *groES* promoter element was indicated in red.

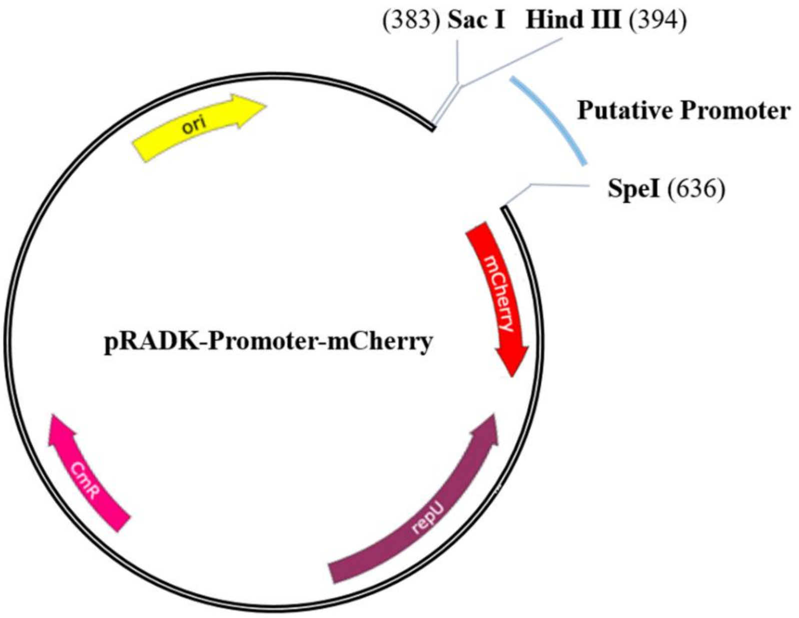

Figure S4. The promoter activity testing vector constructed by enzymatic ligation.

Table S1. Promoter sequences of P1−P24.

Promoters	Sequence (5' to 3')
**P1**	*AGACCATCGGTGTGGACGGCGGGCTGTACCCGCACTGAGCACGGGTGGCTGAACATAGGTGCTGGGCACAGATACCTGCCTGGGAAGCAGCTGGTACGACGTCTCCGTCAGGCATCTGCACACCCCGCAGGCACAGCACCGGCACGGTCTCGAATTGAACCCTGTCCACTCTGGCGCCGCTTCGGCGCGCCACACGTGTAGACTGGCGCAGACTTCAATGCAGGAGGTACCAAGC*
**P2**	*AAGTGTTGGGCGTACCGGCGCGCGCCCTGATCACGCCGCACGCGGCGGTAGGCACCGACGTGGACAAGGAAGAGGACCTGACCCTGGCTGAAGCTCACCTGCCCGAGGCCTGAAACCGGGCTTATAAAACCTGAGTGCTATGCTCAGATTTAAGCGGCTGCACGTGAAAAAAGGCTAACCTGTTGCCCTTGCGCCTACATGCGGGCGTGTGCATACTGTCCCCC*
**P3**	*TGGAATGGGTCCTCCTGGCGCCCTGCTGTCCAGCACCCTGATCAGCCGCGAATTCATCCTGGGACCACGCTGGGGCCTCGTGGCCCTGGCCGCCCTTGGCCTGCTGGCTTTGGCGGCGCTGTGGACC*

CGCCTGCCCCGCACGTTCGCGGAGCCGGGGGCTGCGGCCGT
GAAGGCACCGGCAGGCGGATAAATACCTTTTAAGGAAGGGTGG
TGGGGGAGGCTGTTATGCTGCGGGT

P4

GACGCGCTCGCCGGGCAGGGCGCCGCGCACCAGCACCACGC
CGGACTCGTCACGGGCGAGCCCCAGCCCCCCCGCGACAAGCT
TCTCAATTTCCAGCGTAAGTAATGGCTCGGACATAGATACAGGG
TAGCGCGGCGCGTGAAGAAATGGCCCGGCCGGGCCTCATCCA
CGGTGTCCTCACAAACCGACAGAATCACTGTATGAAACGCACC
ATT

P5

CCGCCCCCAGATCCTGACCGTGCCCAAGATTGATGTAACCGGG
GCGGCCCAGGGCAGCAAGTCCCAATCCCAGACGGGGAGCGTC
AGCAGGTAGCAGGGTGTTGCGCATAAGCCAGTATGGCCTGCCT
ACCTGGCTGTGCCACGCGCGATCACGTCTAGATTTCTGCCGGC
CACGTCAGAATTCCGACAGGAGGTGTGTGAATAATTAGGTCACT
TAAAGAAACCCTTCATGTTCTAGTGACGGGTCCAGGGAGAGTG
T

P6

CAGAGCGGCTCCCAGGCTTCGTGTGCCTCCCACCAGTGCCCG
GCATTGAAGAGCGTCACCCCGAGGCCCAGAGGTCCTGGCCGC
CGCGCGTGGCAGAGTCGGAAGTCACGCTGCTCAGGGTAACCC
CCGCACCTTCCAATGAATACGCCGTTAGGTGGCCCCATGCAG
GACGTAAGCACCGCGGCGTATATTCGTGGC

P7

CTGATGCGGATGTCCTTATGCAGGGCCTCGCCGCTGATGTCGG
TAGGAAAGGTCGCAGTCATGCGTGAAGCCTAACACCATTGGCG
CAACACAATCGTGCGTTTTTGGCACTTTTGACCCTCGCAGCAAA
GGGCTGTTCAGTCAGGGACCTATGAGCCGCTGTTACATTCCTT
GAGTGCAGTTATGTTAAGATAAGAGAC

P8

CTAAGGAAAGCCTCCACATGCCGCATGGCGGCGGAATGAGGT
CATGCTACCTGCCCACTCGCAACAGGCTCGTTCGGCGGTAAGA
CAAATGCAGTTACGCTACGTCGTGCCATCAGCTACGCGTCAGA
GATGAGGCAATCAGACTCATCTCTGAAGGCCAGACTTAGCTGG
TATTTGCGGGACATCCATCCCGTCTTCGCACGCCCTTGAGAAA
GTACTGCTTTGCACATTAGAGGCCCTTGGGGAGTCTCATGCTG
CGCTCAACAACGAAGCAAATTCATCGTTGAGCAAACGACTTGGA
GGAAACGAACATGGGTTGGATTATCACTATTCTG

P9

GCTGGCCATCTCGCGGCAGATCTGCGTGGTCCAGGCTCGCATT
CCGCTGGTGTTGGCTGCCTTCACCCCTCTGCCTCAGTGCCGGC
CAGAAGACTGGGTGAGTTTTCGTACAACAGCCCCACTGCATGG
TTTTGTACTCGGTTGAAAGCTAAAGAACGCTGACAGGCACGGTA

ACCAATCTTTCATTCTCATAGAGATTTAGATAAGATGAATGCCTT
CCT

P10

TTGCTCTTTTCCTGGCAAACAGAATATGTACTGCACGTGATCTTA
CAAGCAGCACCAGGCGAGGTACTTCTCAGCGCCTGGAGTTCG
GGAAACAAAACAAAAAACGTTATCGTGTAATTTCGCTTACAACG
AAAAAAGCTCGTCTACATCACAGAATTTGTCTTGTCAAATCTGAT
TCATCTTTCATTCATATGTAGGTCGTGCTGTTTACACTGCCTGAT

P11

ACCTGCTCTGGGACCGCAGGGCGCCTTCGTGATTCTGCTGGCA
GTCAGCGCCCTGGCCCTGGTTGTTTTTGTGGCCAGCATGCTGC
GCCTGCGGGCCCTGGGGCTCCGGCCCACTTTCGCCTGAAGGC
CCGACGGCGCGCTTAACGCTTCCCTAAGAGGAATGAGCGCCTT
TGTGACCCGCCTGCCGGGCCGGAGTGCCAGGATGAGGCACCA
CAAGGAGACCAC

P12

CGATAAATCCTTTTGGTGGGGCGGTGAGGTCAATCCAGGCAAG
AGCATATCCCGGGCCTGGGCGGCAACGTAGGTGCATCTTACAC
ATGATGCTCACTGTACGTCTAGTTTGACCTTACCGCAGGGACTG
AATGCCCAGGTGTACAAGGTATGCACATAAGCCTGAGCTGAGT
TTCATGTTGTAGACACTCATGATGCGTGCTACGCTGAACACTGA
CGGTCACGCCTGTTCTATGTGTTCAAGCGCGGCCCGCAGACAG
GAGAGGC

P13

TGGATCTTCCTGCCGAGTTGACGTGGTCGAAGGTGTGTTGAAG
TGAGGAGCGTGCCAAAGCTCTGGTGAGCGAGAAAGGGCAGGC
CAGTTGTATATGGAAGGCTTGTGCTACCAACAGTGGTGCACGTT
CTGGGGCCCACTTCTTTCGAGAAAGGTGTCAAGTTGGCGTGGC
TGAATGGGTTTAACTTTGCGACGTTCTCCAAATCTGATCTGCAT
AGGCGGCGGCGCAACAGAAGGTTCAGCTTGTTTCACGGGTGG
CATAAAGATTCACTGCGCATTTTCCTACCTGCGCTGCAGCAACT
ACAGAGCAAGCTGCTGAC

P14

CAACAGCTGCCTTGTGGATTCAGGCGGTTGGTGTATCGGTGAT
TATGGCGTTGACTGTGGTTGCTCTTGGGCATCTTGCGATCGTTG
CTGTAGCAAGGCTCCGCCAGAGCCGCACTCCTTGAGCAGTGG
GCTGCGATAAAGCGTCAAGTTGTCCCCTTCATGGTTGATCCTGC
ACTATCCTGAGCAGGATATGCTCACTGAGCGTTCGCTCAAAGG
GGTGGTTGG

P15

TATCAGTGACGACAGCAATTCCTGGCGTGACACGCTACGCCGG
CGCTTCGGGTCTTCTGATATGCAGCCCGCGCCAATGGTCTTCA
GCAGATCGTGGTTTGTCTGGCTGCCGTGTATAGTGGAAAGCTG
TATGGTGCCCCGTGAGAAGTCAAACGGGCAAGCCGGAGGCAT

AGCTTACCGGTAGCGCCGAAGAGGTTCCCATGATCCTGAATCT
ATTTATTGTTCTGTTCGCCTTGATC

P16

AGCCCGGCATAAACCTCACCGCCGGGACAGTTGATGTACATCT
GGATTTCCTGCTCGGGGTTCTGGGAGTCAAGCAGCAGCAGCTG
CGCGACGATGGTATTGGCCATCTGGGATTCAATGGGAGTCCCC
ACGAAAATAATCCGGTCTTTCAGCAGCCGCGAGTAGATGTCATA
CATCCGCTCACCGCGTCCGGTCTGCTCGATCACGTAGGGAATA
ACGCTCATGGGCGGCATTATCGCATGGGTCAGTGGGAGGAAG
GTGGGCAAGACTGCCCTCAGGTGTAGAGGAGATTGCGGCACTT
GCCGAAAAATTAATGCGGCCCGTTTTCCGCGCCCAGCTCGTCT
ACCCTGAAGGTCCATTTCAATCACAGGAGGTTTGAC

P17

AACGCTCAGGCCTGCACCCTTGACCTCTCGGCGCATCACAGGC
GCACACCGGTCACCTTGTCCGGAGGCAGCCACCTGCCGCCTG
TTCAGGATGAGCGCTATCCGCTGGTCATGGGCAAGTACGACTA
TTACTGGCTGCGCCTGAACTGACGGCAAGCCCAGATTGCTTTA
TGCGGGCTGCCGGCTTAGGCCCGGACAGGTCTTAAGATTCGG
TAGGGAGCCGATTTTTCAGTTTCTCTGGGATGGTGCATCATGG
GATC

P18

CATTAAGGTCTTTGCTGCTCTTCTGATTGGTTCTGTGGTCCTGT
ATCTACTTCGAGAGCCTGCCGTGGCTTTTCTTGTTGGAGAAAAG
TACGCAGCGGCGGCCGATATCATGGCAGTCACAGTCTGGCTGA
TGCCTTTCATCGGGTTCAACACGGTCCTGTTTAATTTCTGGGTG
TTTCCTGCCCGGCGGGATGCAATCGCGAATGCCCTGCTGGTAG
TGAATGCCCTGATTTCGCTGGGTTGGGTGCTATTTATGGCCGG
TCGTACGAATCTACCGCTCTACACGTTCGGTATCTCAGCTGCAG
AAGTCGTTATTAGTGGCGTGCTCATGCTGTTGGTTCTACGAAAT
TTGCGATACCTACTCAGGTTAGAGGTGGCAA

P19

GACCTTGGGAGCAGTGAACGTAGGGGTTGCGGGAGCGCCTGA
GGTGCGTTGTGCACCTGAGTACCCCCCACCAATCAACAGGAGA
GAGAGAGAGAAAGTGAGGATGTTCCTCATGTAATAGTAAGCTC
CTTTACGGTCCCTTTACGGGTGGAAAACGATATGGACGCCTG
ACAGGGTACGGCCTAAGATGATTTTCGTTATGAAGAAGATTCTC
ATGCTG

P20

GCCAGGCAGTCCGCAGCTCTTGACTGAAACCCTGCTGCGCAG
GCTGAGAGGCTGGAGGCTGAACCGGCACGGCAGGCAGGCTA
GCAGAGCCAGCAGCGGTGCAGCACGGACGAGCCTGCCTCCCG
ACAACAAGGTCAGAGTCTTCATGCAGGCTTCAGACTGGGTCGC
GCCGCTGCAGGGAGACAGGTACTCTGACCGGC

**P21**	*AGAGAACTCCAAGAGCTTTGCACGCTGGATTGTTCTTATATTCC<br>AGGCAGGCACCCAGCGCCCTTGACACCGCGCTGGTCGCTTTC<br>CCAACCGCTTTTCATACTTTCTTCACAGCCCAGGAAATCTGATTT<br>AGTGTGAAGTTTTTCAATGTTGTGCAGGCAATGACCCGGCAGC<br>ATGTCTAAGCAGCACAAATTTCGTAGGCACACCCGGCGTTGGA<br>GGCGACTCAGACGTCAAATTACGGGTGTCCTGCATGGAGACTT*
**P22**	*GCAGTTGCCCCGAAAGTGCCTTGGGCGCTTTCCTGCCCCTCCC<br>TCTGCATACGCTCTCTGCCGCCTTTTCGCAGTTCGTCTCAAGCT<br>TGGCACAGGACCCTCATATGAGGCAGATGGCCGGGCCGTGGG<br>GAACATCAGCCCCCACCATTCCAATCATTAAGATAAGACAAAGA<br>ATAATGGGGTAAATTCCCCGCACGACT*
**P23**	*CTGTGGTGGGCCGGCACGCCGTCATCAGTGCTCCCAGCGACG<br>CTGGCCTGCAACTTGTGATCGGCGACCGCAGCGTCGTGCGGC<br>TGTAGTGGGCAGAGGTTCGTCTGATCATGAGCTGCTCCGGTAT<br>GAACGCTCCGATTGCGCCCCGGTCTTGAGCGGACGCACACCC<br>GCCTGGGCTCAGGGCCCACTACCCTGGGCGC*
**P24**	*GTCCGGGTACAGGACTTCCAGGGCCGAGAGCACCTGTGGAGC<br>GCGCGTTCTGGCGCCGGCAGGAAGGCGAGCGGTCTGGGGCT<br>TGCGGGTCACGCGCCGCAGCCTACCCTGCTGCCGTTCAGGA<br>GACAGTAAGCCGTCCGGCCTAGTCCCGCTGGGCTGGCCAGGG<br>AACCGCCACGCCCGCACAAGCGTAGAGAGAGGT*

Table S2. Classification of the promoter activities.

Promoters	Relative activity
**P8**	+
**P1**	+
**P21**	+
**P2**	**O**
**P5**	**-**
**P6**	**-**
**P7**	**-**
**P10**	**-**
**P11**	**-**
**P15**	**-**
**P19**	**-**
**P22**	**-**
**P23**	**-**
**P13**	**--**

**P16**	**--**
**P17**	**--**
**P3**	**---**
**P9**	**---**
**P14**	**---**
**P18**	**---**

* Promoter activity classification compared with the control promoter PG: +, strong promoters. O, similar activity. -, relatively weak promoters. --, weak promoters. ---, very weak promoters.

Table S3. Primers used to amplify P1−P24.

Primer	Sequence (5' to 3')
P1-F	cccAAGCTTAGACCATCGGTGTGGACGGCGG
P1-R	gACTAGTGCTTGGTACCTCCTGCATTGAAGTCTGCG
P2-F	cccAAGCTTAAGTGTTGGGCGTACCGGCGC
P2-R	gACTAGTGGGGGACAGTATGCACACGCCCG
P3-F	cccAAGCTTTGGAATGGGTCCTCCTGGCGCC
P3-R	gACTAGTACCCGCAGCATAACAGCCTCCCC
P4-F	cGAGCTCGACGCGCTCGCCGGGCAGG
P4-R	gACTAGTAATGGTGCGTTTCATACAGTGATTCTGTCGGTTTGTG
P5-F	cccAAGCTTCCGCCCCCAGATCCTGACCGTG
P5-R	gACTAGTACACTCTCCCTGGACCCGTCACTAG
P6-F	cccAAGCTTCAGAGCGGCTCCCAGGCTTCGT
P6-R	gACTAGTGCCACGAATATACGCCGCGGTGC
P7-F	cccAAGCTTCTGATGCGGATGTCCTTATGCAGGGCCT
P7-R	gACTAGTGTCTCTTATCTTAACATAACTGCACTCAAGGAATGTAACAGCGG
P8-F	cccAAGCTTCTAAGGAAAGCCTCCACATGCCGCATG
P8-R	gACTAGTCAGAATAGTGATAATCCAACCCATGTTCGTTTCCTCCAA
P9-F	cccAAGCTTGCTGGCCATCTCGCGGCAGATCTG
P9-R	gACTAGTAGGAAGGCATTCATCTTATCTAAATCTCTATGAGAATGAAAGATTGGTTA
P10-F	cccAAGCTTTTGCTCTTTTCCTGGCAAACAGAATATGTACTGCACG
P10-R	gACTAGTATCAGGCAGTGTAAACAGCACGACCTACAT
P11-F	cccAAGCTTACCTGCTCTGGGACCGCAGGG
P11-R	gACTAGTGTGGTCTCCTTGTGGTGCCTCATCCTGG
P12-F	cccAAGCTTCGATAAATCCTTTTGGTGGGGCGGTGAGGT
P12-R	gACTAGTGCCTCTCCTGTCTGCGGGCCG
P13-F	cccAAGCTTTGGATCTTCCTGCCGAGTTGACGTGGT
P13-R	gACTAGTGTCAGCAGCTTGCTCTGTAGTTGCTGCAG

P14-F	cccAAGCTTCAACAGCTGCCTTGTGGATTCAGGCG
P14-R	gACTAGTCCAACCACCCCTTTGAGCGAACGC
P15-F	cccAAGCTTTATCAGTGACGACAGCAATTCCTGGCGTGA
P15-R	gACTAGTGATCAAGGCGAACAGAACAATAAATAGATTCAGGATCATGGG
P16-F	cccAAGCTTAGCCCGGCATAAACCTCACCGCC
P16-R	gACTAGTGTCAAACCTCCTGTGATTGAAATGGACCTTCAGGGT
P17-F	cccAAGCTTAACGCTCAGGCCTGCACCCTTGAC
P17-R	gACTAGTGATCCCATGATGCACCATCCCAGAGAAACTGA
P18-F	cccAAGCTTCATTAAGGTCTTTGCTGCTCTTCTGATTGGTTCTGTGGT
P18-R	gACTAGTTTGCCACCTCTAACCTGAGTAGGTATCGCA
P19-F	cccAAGCTTGACCTTGGGAGCAGTGAACGTAGGGGT
P19-R	gACTAGTCAGCATGAGAATCTTCTTCATAACGAAAATCATCTTAGGCCGT
P20-F	cccAAGCTTGCCAGGCAGTCCGCAGCTCTT
P20-R	gACTAGTGCCGGTCAGAGTACCTGTCTCCCTGC
P21-F	cccAAGCTTAGAGAACTCCAAGAGCTTTGCACGCTGG
P21-R	gACTAGTAAGTCTCCATGCAGGACACCCGTAATTTGACG
P22-F	cGAGCTCGCAGTTGCCCCGAAAGTGCCTTGG
P22-R	gACTAGTAGTCGTGCGGGGAATTTACCCCATTATTCTTTGT
P23-F	cccAAGCTTCTGTGGTGGGCCGGCACGC
P23-R	gACTAGTGCGCCCAGGGTAGTGGGCCC
P24-F	cccAAGCTTGTCCGGGTACAGGACTTCCAGGGCC
P24-R	gACTAGTACCTCTCTCTACGCTTGTGCGGGCG

*Lowercase letters indicate protected bases, and underscores indicate enzyme cleavage sites.

Table S4. Primers used for homologous recombination.

**Primer**	**Sequence (5' to 3')**
P8-H-F	gctcgaattctagaagcttCTAAGGAAAGCCTCCACATGCC
P8-H-R	caatcctgtagacttgcgcatCAGAATAGTGATAATCCAACCCATGTTCG
P1-H-F	cgttgtccatgggacgctaaAGACCATCGGTGTGGACGGC
P1-H-R	ctgcgactcagtcgtgccatGCTTGGTACCTCCTGCATTGAAGTCTG
P21-H-F	caactccgatcagtctctagAGAGAACTCCAAGAGCTTTGCACGCTG
P21-H-R	gacttcgtcatatttgacatAAGTCTCCATGCAGGACACCCGT
P2-H-F	gatttcgcctatgacctgaAAGTGTTGGGCGTACCGGCG
P2-H-R	gagcaacgattcttttcatGGGGGACAGTATGCACACGCCC
P22-H-F	cgcgcccgaaccggagtaaGCAGTTGCCCCGAAAGTGCCTTG
P22-H-R	cgtcggtaatactattcatAGTCGTGCGGGGAATTTACCCCA
SAVE-1-F	aagcttctagaattcgagctcccgggtac
SAVE-1-R	atgcgcaagtctacaggattgctgctaac

SAVE-2-F	ttagcgtcccatggacaacgtattcacg
SAVE-2-R	atggcacgactgagtcgcagag
SAVE-3-F	ctagagactgatcggagttgcgccgatatagc
SAVE-3-R	atgtcaaatatgacgaagtcgcctaaacgccaattg
SAVE-4-F	tcaggtcataggcgaaatcatcatgtccgc
SAVE-4-R	atgaaaagaatcgttgctctgttaagcacgttgc
SAVE-5-F	ttactccggttcgggcgcgtcttc
SAVE-5-R	atgaatagtattaccgacgatcgactcgtcttggc
